# Protocol optimization for enhanced production of pigments in *Spirulina*

**DOI:** 10.1007/s40502-013-0045-8

**Published:** 2013-10-12

**Authors:** Devendra Kumar, Neeraj Kumar, Sunil Pabbi, Suresh Walia, Dolly Wattal Dhar

**Affiliations:** 1 Centre for Conservation and Utilisation of Blue Green Algae, Indian Agricultural Research Institute, New Delhi, 110012 India; 2 Department of Microbiology, Kurukshetra University, Kurukshetra, India; 3 Division of Agricultural Chemicals, Indian Agricultural Research Institute, New Delhi, India

**Keywords:** Mass production, Pigments, *Spirulina*

## Abstract

*Spirulina* has attracted special attention due to its importance as human foodstuff and natural colours with specific functional properties. These functional properties have been attributed to phycobilins, carotenoids, phenolics and unsaturated fatty acids. Present study was conducted under controlled phytotron conditions to identify the efficient strains of *Spirulina* in terms of pigment synthesis and to optimize their enhanced production. Methodology for enhanced production was standardized by varying specific environmental parameters (light intensity, temperature, carbon dioxide concentration, pH and NaCl level). Different strains of *Spirulina* depicted variability and environmental parameters showed distinct influence on pigments. Growth and pigment production was recorded to be most efficient under optimized conditions of light intensity (70 μmol m^−2^ s^−1^), temperature (30 °C), CO_2_ concentration (550 ppm and 750 ppm), pH (10.5) and NaCl level (2 g L^−1^).

*Spirulina*, a cyanobacterial genus has been reported to be the complete organic food source and contains high proteins with well balanced amino acids. It is also rich in carbohydrates, vitamins, minerals, phenolics, pigments (chlorophyll, carotenoids and phycobilins) and poly-unsaturated fatty acids (Miranda et al. 1998; Anupama 2000). It is successfully used in aquaculture and poultry industries (Belay et al. 1993; Estrada et al. 2001). This genus is reported to be photosynthetic, filamentous in an open left-hand helix, with predominance from aquatic habitats having high levels of carbonates, bicarbonates and alkaline pH (up to 11). The large scale production of S*pirulina* depends on nutrient availability, temperature, light, pH and CO_2_ concentration. In view of this, a study was conducted to optimize the protocol for enhanced production of pigments in *Spirulina*. Ten strains procured from the culture collection of Centre for Conservation and Utilisation of Blue-Green Algae (CCUBGA), IARI, New Delhi were grown in chemically defined Z-medium, which is rich in carbonate and thus, has successively served as a common culture medium (Zarrouk 1966). *Spirulina* strains were grown under diffused light intensity of 55 μmol m^−2^ s^−1^ at 28 °C ± 2 °C temperature with 16:8 h light: dark cycle. Initial biomass of 0.1 g L^−1^ (Costa et al. 2000) was taken as inoculum in 100 mL medium in conical flasks. The influence of different pH (9.5, 10.5, 11.5, 12.5, 13.5) and NaCl concentrations (0, 0.5, 1.0, 1.5, 2.0 g L^−1^) was studied on the pigment production under culture room facility. Studies were also conducted under varying temperature (25, 30 and 35 °C), light intensity (55, 70 and 85 μmol m^−2^ s^−1^) and CO_2_ concentration (350, 550 and 750 ppm) in growth chambers of National Phytotron Facility, IARI. Homogenized suspension was drawn at 15th day of incubation for the estimation of chlorophyll (Lichtenthaler 1987), carotenoids (Jensen 1978) and phycobiliproteins (Bennett and Bogorad 1993).

Most of the strains exhibited uniform suspension in the medium and some showed planktonic growth behaviour. Difference in the thallus colour observed is reported to be under the control of chlorophyll to phycobilin ratio. Pigments can also be subjected to oxidation by molecular oxygen, which is known to be a potent inhibitor of microbial processes including photosynthesis (Paerl and Pinckney 1996). The environmental variables can influence growth and pigment production which in turn may change the composition of the biomass by affecting the metabolism. Varied light intensity exhibited differential influence on the accumulation of chlorophyll, carotenoids and phycobilins. Results indicated that a light intensity of 55 μmol m^−2^ s^−1^ was optimum for maximum chlorophyll and that of 85 μmol m^−2^ s^−1^ was inhibitory in most cases, except in case of CCC538. Variation in the temperature influenced the pigment profile with 35 °C being the optimum for enhanced chlorophyll. Danesi et al. (2001) reported highest biomass production at 30 °C temperature. High temperatures are the key factors for large scale *Spirulina* cultures outdoors with the optimal temperature reported in range of 35–38 °C (Zarrouk 1966; Hu 2004). The temperature below 20 °C and above 40 °C retarded the growth considerably (Borowitzka and Borowitzka 1988). The highest biomass at 30 °C than at 35 °C may be due to higher partial pressure of CO_2_ in the medium leading to enhanced concentration of bicarbonates with consequent increase in the rate of photosynthesis (Colla et al. 2004). Further, a longer dark cycle respiratory activity in which cells use reserve material for respiration accordingly can decrease the cell weight (Vonshak et al. 1982). In general, chlorophyll content enhanced with increase in carbon dioxide concentration and a concentration of 750 ppm was optimum. Effect of pH was distinct with 10.5 pH being the optimum for enhanced chlorophyll production as has also been reported by Richmond and Grobbelaar (1986). *Spirulina* requires relatively high pH between 9.5 and 9.8 (Hu 2004), which inhibit the contamination by most algae in the culture. Therefore, high amounts of sodium bicarbonate is essential in the culture medium to sustain high pH. High alkalinity is mandatory for the growth and bicarbonate is used to maintain the high pH (Vonshak and Tomaselli 2000). The significant pigment production may be the result of an increase in pH due to the formation of a CO_2_/H_2_CO_3_/HCO^3−^/CO_3_^−2^ system, which may function as useful buffer system for maintaining the alkaline pH. This is important for the optimum growth and helps to prevent the carbon depletion (Richmond 1990; Vonshak 1997). Sodium chloride concentration of 2 g L^−1^ depicted highest chlorophyll, however, several species of *Spirulina* exhibit inhibition of growth in hostile environments of saline lakes (Henrikson 1994). Exposure of *S. platensis* to an enhanced sodium concentration resulted in a 30 % increase in intracellular accumulation of Na^+^ accompanied by small changes in dry mass and chlorophyll content (Kavita and Mohanty 2000) (Table 1). Carotenoids and phycobiliproteins were highest at 70 μmol m^−2^ s^−1^ light intensity and 10.5 pH was optimum for higher carotenoids and phycobiliproteins. In conventional cultivation, light absorption is proportional to the phycocyanin and chlorophyll content of the cells and a slight reduction in phycocyanin may be accompanied by an increase in chlorophyll (Cornet et al. 1992). Carotenoids were maximum at 35 °C temperature and enhanced CO_2_ resulted in their increase with 750 ppm to be an optimum concentration. Sodium chloride concentration of 1.5 g L^−1^ showed highest carotenoids. Phycobiliproteins enhanced significantly at 35 °C in some cultures, whereas decline was observed in others. Enhancement in CO_2_ concentration increased the phycobiliproteins, and sodium chloride concentration of 2 g L^−1^ was most suitable for phycobiliprotein production (Tables 2, 3). Study clearly showed that the pigments from *Spirulina*, which have the potential applications in biotechnology can be enhanced by manipulating cultural conditions and environmental variables. Standardized protocol for enhanced production of pigments involved an optimized light intensity: 70 μmol m^−2^ s^−1^ for chlorophyll and carotenoids, and 85 μmol m^−2^ s^−1^ for phycobiliproteins, temperature: 35 °C for chlorophyll and phycobiliproteins, and 25 °C for carotenoids, CO_2_ concentration: 500 ppm and 750 ppm, pH: 10.5 and NaCl concentration: 2 g L^−1^.

**Table 1:** Influence of environmental variables on chlorophyll (mg g^−1^ dry weight) content of *Spirulina* strains

Environmental variables	*Spirulina* strains^a^										SE (m)±	CD (*P* = 0.05)
	CCC477	CCC478	CCC479	CCC480	CCC481	CCC482	CCC483	CCC538	CCC539	CCC540		
Light (μmol m^−2^ s^−1^)												
**55**	1.550	1.934	1.795	2.611	1.445	1.781	3.188	1.725	2.148	2.553	0.20	0 .41
**70**	1.192	0.924	0.611	1.359	1.245	2.341	2.783	2.156	1.808	2.358	0.23	0 .47
**85**	1.014	1.250	1.143	1.304	1.227	1.620	1.298	2.134	1.548	1.991	0.14	0.29
Temperature (°C)												
**25**	0.408	0.587	0.907	1.513	1.147	1.626	1.293	1.650	0.919	1.693	0.16	0.33
**30**	0.640	0.859	1.103	1.133	1.294	0.932	1.416	1.388	1.157	3.252	1.29	2.63
**35**	1.416	0.992	1.542	1.848	1.444	2.238	1.916	2.992	1.875	1.804	0.24	0.50
CO_2_ concentration (ppm)												
**350**	2.547	2.648	1.257	2.008	0.973	0.665	2.324	1.314	1.235	0.858	0.27	0.56
**550**	0.745	0.979	1.225	1.052	1.339	2.176	2.576	2.150	2.074	2.160	0.24	0.50
**750**	3.163	4.555	4.035	4.320	2.952	5.961	1.140	3.861	3.056	4.466	0.40	0.82
pH												
**9.5**	1.632	1.207	1.134	2.062	1.365	0.762	1.019	1.352	1.617	0.953	0.27	0.56
**10.5**	1.752	2.783	1.579	4.412	2.641	2.466	2.206	2.593	1.527	2.141	0.32	0.66
**11.5**	1.648	1.787	0.959	1.334	1.552	0.731	0.530	0.459	0.486	0.611	0.07	0.15
**12.5**	1.369	1.902	0.447	0.965	0.771	0.268	0.579	0.359	0.529	0.193	0.11	0.24
**13.5**	0.318	1.859	0.993	0.421	1.285	0.442	0.114	0.153	0.187	0.198	0.22	0.45
NaCl concentration (g L^−1^)												
**0**	0.620	0.872	1.243	0.563	0.661	0.531	0.234	0.638	0.566	0.877	0.02	0.05
**0.5**	0.981	0.457	0.936	0.782	1.835	0.657	0.373	0.668	0.764	2.065	0.04	0.08
**1**	1.371	1.749	0.357	0.702	0.349	1.860	0.468	0.749	0.463	1.662	0.01	0.02
**1.5**	0.830	1.348	0.574	0.878	0.654	1.842	0.665	0.645	0.848	1.325	0.02	0.05
**2**	2.252	1.247	1.562	1.137	1.374	0.481	0.648	0.647	0.704	1.638	0.02	0.04

^a^CCC477, CCC478, CCC479, CCC480-*Spirulina platensis*, CCC481-*Spirulina maxima*, CCC482-*Spirulina lonar*, CCC483-*Spirulina platensis* (mutant), CCC538, CCC539-*Arthrospira* sp., CCC540-*Spirulina* sp.

**Table 2:** Influence of environmental variables on carotenoids (mg g^−1^ dry weight) content of *Spirulina* strains

Environmental variables	*Spirulina* strains^a^										SE (m)±	CD (*P* = 0.05)
	CCC477	CCC478	CCC479	CCC480	CCC481	CCC482	CCC483	CCC538	CCC539	CCC540		
Light (μmol m^−2^ s^−1^)												
**55**	0.243	0.306	0.257	0.287	0.141	0.193	0.268	0.133	0.222	0.266	0.01	0.03
**70**	0.147	0.144	0.094	0.228	0.151	0.307	0.307	0.233	0.204	0.271	0.02	0.05
**84**	0.250	0.246	0.202	0.179	0.106	0.231	0.193	0.170	0.202	0.169	0.02	0.04
Temperature (°C)												
**25**	0.123	0.073	0.157	0.301	0.190	0.235	0.231	0.184	0.252	0.228	0.02	0.05
**30**	0.149	0.285	0.184	0.186	0.143	0.176	0.146	0.173	0.179	0.208	0.02	0.05
**35**	0.206	0.224	0.280	0.148	0.166	0.184	0.129	0.181	0.149	0.158	0.02	0.05
CO_2_ concentration (ppm)												
**350**	0.133	0.244	0.180	0.290	0.188	0.216	0.235	0.158	0.181	0.208	0.01	0.03
**550**	0.159	0.154	0.241	0.393	0.244	0.222	0.274	0.244	0.214	0.203	0.02	0.04
**750**	0.269	0.208	0.179	0.229	0.263	0.203	0.360	0.312	0.224	0.304	0.03	0.06
pH												
**9.5**	0.144	0.200	0.358	0.140	0.109	0.114	0.117	0.180	0.183	0.121	0.02	0.04
**10.5**	0.233	0.292	0.331	0.283	0.203	0.185	0.306	0.269	0.217	0.182	0.05	0.11
**11.5**	0.180	0.259	0.261	0.073	0.069	0.111	0.107	0.069	0.122	0.112	0.02	0.04
**12.5**	0.169	0.285	0.166	0.048	0.090	0.030	0.080	0.045	0.124	0.063	0.02	0.05
**13.5**	0.055	0.152	0.157	0.042	0.013	0.019	0.005	0.003	0.037	0.013	0.01	0.03
NaCl concentration (g L^−1^)												
**0**	0.079	0.074	0.045	0.094	0.034	0.141	0.044	0.040	0.100	0.103	0.08	0.02
**0.5**	0.137	0.136	0.057	0.081	0.057	0.230	0.093	0.045	0.096	0.151	0.09	0.03
**1**	0.034	0.073	0.083	0.048	0.080	0.105	0.036	0.074	0.077	0.056	0.07	0.01
**1.5**	0.030	0.133	0.114	0.024	0.123	0.105	0.029	0.125	0.120	0.017	0.05	0.01
**2**	0.042	0.010	0.020	0.034	0.021	0.014	0.019	0.018	0.035	0.030	0.03	0.08

^a^CCC477, CCC478, CCC479, CCC480- *Spirulina platensis*, CCC481-*Spirulina maxima*, CCC482-*Spirulina lonar*, CCC483-*Spirulina platensis* (mutant), CCC538, CCC539-*Arthrospira* sp., CCC540-*Spirulina* sp.

**Table 3:** Influence of environmental variables on phycobiliproteins (mg g^−1^ dry weight) content of *Spirulina* strains

Environmental variables	*Spirulina* strains^a^										SE(m)±	CD (*P* = 0.05)
	CCC477	CCC478	CCC479	CCC480	CCC481	CCC482	CCC483	CCC538	CCC539	CCC540		
Light (μmol m^−2^ s^−1^)												
**55**	1.341	1.160	4.059	3.723	1.558	2.091	1.817	1.111	2.277	1.603	0.09	0.19
**70**	0.354	0.569	2.325	2.194	1.749	4.650	6.235	4.091	3.574	4.922	0.36	0.75
**84**	0.785	0.614	2.798	3.030	1.703	4.035	3.300	5.134	5.540	3.622	0.15	0.30
Temperature (°C)												
**25**	1.153	1.051	5.175	3.151	2.199	1.663	1.278	1.431	0.599	0.731	0.13	0.28
**30**	0.881	2.333	3.063	3.713	2.660	4.803	4.497	3.283	3.247	2.708	0.38	0.78
**35**	6.651	3.321	11.746	1.766	8.199	2.346	4.489	3.722	0.955	2.627	0.38	0.78
CO_2_ concentration (ppm)												
**350**	7.266	7.524	4.731	8.557	2.239	1.367	5.178	3.431	2.938	1.848	0.10	0.20
**550**	3.036	4.255	10.505	2.930	9.699	4.954	4.639	4.866	7.979	4.483	0.09	0.03
**750**	4.562	6.918	6.805	8.089	7.582	8.455	1.848	6.275	2.477	8.400	0.09	0.02
pH												
**9.5**	5.023	2.908	2.244	7.183	11.243	0.995	4.462	4.418	9.796	2.485	0.14	0.24
**10.5**	8.603	3.922	6.318	20.266	5.985	8.995	12.197	15.536	5.126	9.219	0.33	O.66
**11.5**	2.789	1.818	2.675	2.265	1.760	2.278	4.156	2.705	2.169	3.200	0.23	0.45
**12.5**	3.635	1.118	1.932	1.575	1.752	1.150	2.915	1.520	1.538	0.739	0.22	0.56
**13.5**	1.274	2.113	0.754	0.619	1.043	0.872	0.822	0.331	0.806	0.664	0.19	0.34
NaCl concentration (g L^−1^)												
**0**	3.192	3.796	2.889	2.614	5.782	4.390	3.640	2.946	2.592	2.836	0.07	0.01
**0.5**	3.697	5.951	6.206	4.583	5.976	6.425	6.306	5.206	4.545	5.937	0.13	0.29
**1**	5.122	4.622	5.625	4.236	5.713	6.765	4.913	6.931	5.929	5.390	0.15	0.34
**1.5**	6.349	5.613	5.277	6.512	8.514	6.636	5.475	6.310	6.600	6.798	0.19	0.34
**2**	6.492	6.611	11.486	5.739	6.764	4.259	5.512	10.323	11.792	4.629	0.09	0.20

^a^CCC477, CCC478, CCC479, CCC480- *Spirulina platensis*, CCC481-*Spirulina maxima*, CCC482-*Spirulina lonar*, CCC483-*Spirulina platensis* (mutant), CCC538, CCC539-*Arthrospira* sp., CCC540-*Spirulina* sp.

## References

[CR1] Anupama PR (2000). Value-added food: Single cell protein. Biotechnology Advances.

[CR2] Belay A, Ota Y, Miyakawa K, Shimamatsu H (1993). Current knowledge on potential health benefits of *Spirulina*. Journal of Applied Phycology.

[CR3] Bennett A, Bogorad L (1993). Complementary chromatic adaptation in a filamentous blue green alga. Journal of Cell Biology.

[CR4] Borowitzka MA, Borowitzka LJ (1988). Microalgal biotechnology.

[CR5] Colla LM, Reinehr CO, Reichert C, Costa JAV (2004). Production of biomass and nutraceutical compounds by *Spirulina platensis* under different temperature and nitrogen regimes. Bioresource Technology.

[CR6] Cornet JF, Dussa CG, Cluzel P, Dubertret GA (1992). Structured model for simulation of cyanobacterium *Spirulina platensis* in photobioreactors: Identification of kinetic parameters under light and mineral limitations. Biotechnology and Bioengineering.

[CR7] Costa JAV, Linde GA, Atala DIP, Mibieli GM, Kruger RT (2000). Modelling of growth conditions for cyanobacterium *Spirulina platensis* in microcosms. World Journal of Microbiology & Biotechnology.

[CR8] Danesi, E. D. G., Rangel, C. O., Pelzer, L. H., Carvalho, J. C. M., Sat, S. & Moraes, I. O. (2001). Production of *Spirulina platensis* under different temperatures and urea feeding regimes for chlorophyll attainment. *Proceedings: Eighth International Congress in Engineering and Food* (Vol. 2 In: 1978–1982).

[CR9] Estrada JE, Bescos P, Villar Del Fresno AM (2001). Antioxidant activity of different fractions of *Spirulina platensis* protein extract. Il Farmaco.

[CR10] Henrikson R (1994). Microalga *Spirulina*-Superalimento del future.

[CR11] Hu Q, Richmond A (2004). Industrial production of microalgal cell mass and secondary products-major industrial species: *Arthrospira (Spirulina) platensis*. Handbook of microalgal culture: Biotechnology and applied phycology.

[CR12] Jensen, A. (1978). Chlorophylls and carotenoids. In J. A. Hellebust & J. S. Caraig (Eds.), *Phycological methods: Physiological and biochemical methods* (pp. 59–70). Cambridge: Cambridge University Press.

[CR13] Kavita V., Mohanty P. (2000). Alterations in the structure of phycobilisomes of the cyanobacterium *Spirulina platensis* in response to enhanced Na^+^ level. World Journal of Microbiology & Biotechnology.

[CR14] Lichtenthaler, H. K. (1987). Chlorophyll and carotenoids: pigments of photosynthetic biomembranes. In L. Paker & R. Douce (Eds.), *Methods in enzymology*, (Vol. 148, pp. 350–382). Boca Raton: CRC Press.

[CR15] Miranda MS, Cintra RG, Barros SBM, Filho JM (1998). Antioxidant activity of the microalga *Spirulina maxima*. Brazilian Journal of Medical and Biological Research.

[CR16] Paerl HW, Pinckney JL (1996). A minireview of microbial consortia: Their role in aquatic production and biogeochemical cycling. Microbial Ecology.

[CR17] Richmond A (1990). Handbook of microalgal mass culture.

[CR18] Richmond A, Grobbelaar JU (1986). Factors affecting the output rate of *Spirulina platensis* with reference to mass cultivation. Biomass.

[CR19] Vonshak, A. (1997). *Spirulina*: Growth, physiology and biochemistry. In A. Vonshak (Ed.), *Spirulina platensis (Arthrospira)*: *Physiology*, *cell-biology and biotechnology* (pp. 43–65). London: Taylor & Francis.

[CR20] Vonshak A, Abeliovich A, Boussiba S, Arad S, Richmond A (1982). Production of *Spirulina* biomass: Effects of environmental factors and population density. Biomass.

[CR21] Vonshak, A., & Tomaselli, L. (2000). *Arthrospira* (*Spirulina)*: Systematics and ecophysiology. In A. Whitton & E. Potts (Eds.), *The ecology of cyanobacteria* (pp. 505–522). The Netherlands: Kluwer Academic Publishers.

[CR22] Zarrouk, C. (1966). Contribution a I’ etude d’ une cyanobacterie: Influence de divers facteurs physiques et chimiques et la photosynthese de *Spirulina maxima* (Setchell et Gardener) Geitler. Ph.D. Thesis, University of paris, France.

